# Erratum to: Effect of insertion route on risk of central line-associated bloodstream infection in critically ill patients

**DOI:** 10.1186/s13054-015-1001-y

**Published:** 2015-09-04

**Authors:** N. Hassan, A. Hammodi, R. Alhubail, Ali Alazem, N. Rayyan

**Affiliations:** ICU department, King Fahad Specialist Hospital-Dammam, 6830 Ammar Bin Thabit St, Al Muraikabat, 32253-3202 Dammam, Saudi Arabia

## Erratum

After publication of our recent Poster presentation [1], we noticed that three authors were inadvertently omitted as co-authors. The author list is now complete and reads follows: N. Hassan, A. Hammodi, R. Alhubail*, Ali Alazem, N. Rayyan.
